# Management of Cytomegalovirus Infections in the Era of the Novel Antiviral Players, Letermovir and Maribavir

**DOI:** 10.3390/idr16010005

**Published:** 2024-01-18

**Authors:** Jocelyne Piret, Guy Boivin

**Affiliations:** Centre de Recherche en Infectiologie, CHU de Québec-Université Laval, Quebec City, QC G1V 4G2, Canada; jocelyne.piret@crchudequebec.ulaval.ca

**Keywords:** cytomegalovirus, immunocompromised patients, antiviral drugs, drug resistance, letermovir, maribavir

## Abstract

Cytomegalovirus (CMV) infections may increase morbidity and mortality in immunocompromised patients. Until recently, standard antiviral drugs against CMV were limited to viral DNA polymerase inhibitors (val)ganciclovir, foscarnet and cidofovir with a risk for cross-resistance. These drugs may also cause serious side effects. This narrative review provides an update on new antiviral agents that were approved for the prevention and treatment of CMV infections in transplant recipients. Letermovir was approved in 2017 for CMV prophylaxis in CMV-seropositive adults who received an allogeneic hematopoietic stem cell transplant. Maribavir followed four years later, with an indication in the treatment of adult and pediatric transplant patients with refractory/resistant CMV disease. The target of letermovir is the CMV terminase complex (constituted of pUL56, pUL89 and pUL51 subunits). Letermovir prevents the cleavage of viral DNA and its packaging into capsids. Maribavir is a pUL97 kinase inhibitor, which interferes with the assembly of capsids and the egress of virions from the nucleus. Both drugs have activity against most CMV strains resistant to standard drugs and exhibit favorable safety profiles. However, high-level resistance mutations may arise more rapidly in the *UL56* gene under letermovir than low-grade resistance mutations. Some mutations emerging in the *UL97* gene under maribavir can be cross-resistant with ganciclovir. Thus, letermovir and maribavir now extend the drug arsenal available for the management of CMV infections and their respective niches are currently defined.

## 1. Introduction

The Herpesviridae forms a large family of DNA viruses. This family includes three subfamilies, the *α-herpesvirinae* (herpes simplex viruses 1 and 2 and varicella zoster virus), the *β-herpesvirinae* (cytomegalovirus (CMV), human herpes viruses 6A, 6B and 7) and the *γ-herpesvirinae* (Epstein–Barr virus and human herpes virus 8) [1]. These viruses are ubiquitous and their epidemiology is not associated with seasonal variations. They cause a large spectrum of diseases, the severity of which is markedly dependent upon the host’s immune status. All these viruses have the ability to establish a life-long latency in different cell types and to cause recurrent infections upon reactivation of latent virus.

CMV can be transmitted by close contact with saliva, urine, genital secretions and blood as well as following organ transplantation [2]. The seroprevalence of CMV in the population ranges from 50 to 90% [2]. CMV can cause a primary infection, recurrent episodes following the reactivation of a latent virus or a new infection with another viral isolate (superinfection or reinfection) [2]. In immunocompetent individuals, CMV infections are generally asymptomatic or present as a mild flu-like febrile illness. However, in immunocompromised patients, it can cause life-threatening tissue-invasive diseases affecting different organs such as the lungs, the gastrointestinal tract, the liver, the eyes and the central nervous system or manifest as a systemic syndrome [2]. Vertical transmission of CMV can also occur in utero, during vaginal delivery and by breast milk [2]. In developed countries, it is estimated that 0.2% to 0.6% of newborns are diagnosed with congenital CMV infection [2]. Clinical manifestations include rash, hepatosplenomegaly, microcephaly and intracerebral calcification [2]. Most clinically diagnosed newborns (90%) will survive the infection, but half of them will suffer unilateral or bilateral sensorineural hearing loss with or without developmental delays [3]. Furthermore, up to 14% of asymptomatic newborns could develop hearing loss or learning problems at later times [3].

In transplant recipients, CMV can induce direct and indirect effects (such as increased risks of opportunistic infections and acute graft-versus-host disease) and can result in excess morbidity and mortality of patients [4]. Two main strategies are used for the prevention of CMV infection and disease in transplant patients [5]. The first one consists in the administration of a prophylactic antiviral treatment to all at-risk patients. The second one is based on the initiation of a pre-emptive antiviral therapy when the blood viral DNA load reaches a certain threshold. Until recently, viral DNA polymerase inhibitors were the only antiviral agents available for the prevention or treatment of CMV infection and disease [6]. However, these drugs have the same target and are associated with a risk of the emergence of cross-resistance. Furthermore, their administration can result in potentially serious side effects [7,8,9,10]. Due to these limitations, it is important to develop novel CMV compounds that act through different mechanisms of action and demonstrate adequate safety profiles [11]. The discovery of new CMV inhibitors has led to the identification of novel potential targets, the viral terminase complex [12,13] and the viral pUL97 kinase [14,15]. Among those compounds, letermovir (LMV) and maribavir (MBV), have been recently approved by the Food and Drug Administration (FDA). 

The four DNA polymerase inhibitors were approved in a 13-year period with ganciclovir (GCV) in 1988, foscarnet (FOS) in 1991, cidofovir (CDV) in 1996 and the prodrug valganciclovir (VGCV) in 2001. There was then a time lag of more than 15 years before a new era could begin with the approval of the first CMV terminase complex inhibitor, LMV in 2017, and the first pUL97 kinase inhibitor, MBV in 2021. This narrative review provides an update on the use of LMV and MBV for the prevention and treatment of CMV infections in transplant recipients.

## 2. Diagnosis of CMV Infection

The diagnostic test of choice for active CMV infection is based on the determination of the viral DNA load in blood samples by quantitative PCR [5]. Several molecular platforms (such as Artus CMV RGQ MDX kit (Qiagen), Cobas AmpliPrep/Cobas Taqman CMV test (Roche) and RealTime CMV molecular test (Abbott)) have been developed and approved by the FDA. Furthermore, an international reference standard has been validated by the World Health Organization to limit inter-laboratory variability [16]. Viral replication can also occur in anatomical compartments in the absence of viremia, especially in cases of CMV-induced gastrointestinal disease, pneumonia and encephalitis. It can thus be useful to determine the viral DNA load in specific body compartments (e.g., gastrointestinal biopsies, bronchoalveolar lavages and cerebrospinal fluid) [5,17,18]. 

As these quantitative PCR assays are very sensitive, antiviral treatment based solely on the determination of viral DNA load could lead to prolonged and unneeded drug exposure. It is thus suggested that the viral load quantification could be complemented with CMV immune monitoring, which is used as a proxy of the ability of the host immune response to control the viral infection [5,19]. Commercially available tests (such as QuantiFERON-CMV, T-Track and T-Spot.CMV) are based on the detection of interferon-γ released by CD4^+^ and/or CD8^+^ T cells following stimulation with CMV-specific antigens or peptides [19,20]. Several studies in solid organ transplant (SOT) and hematopoietic stem cell (HSC) recipients have shown that patients with high CMV-specific immunity had reduced peaks of viral load, higher rates of viral clearance and lower rates of viral reactivation than those who responded weakly [21,22,23,24,25,26,27,28]. CMV immune monitoring combined with viral load quantification may thus help to predict the risk of active CMV infection after transplantation or after antiviral prophylaxis [20]. Such a strategy could also be used to predict the need for secondary prophylaxis or the risk of CMV relapse after treatment [20]. It is thus anticipated that the combined determination of CMV-specific immunity and viremia could guide the use of antivirals for a specific patient allowing a personalized management of CMV infection. However, further investigations are still needed before these tests can be fully implemented in clinical practice. 

## 3. DNA Polymerase Inhibitors

**Figure 1:**
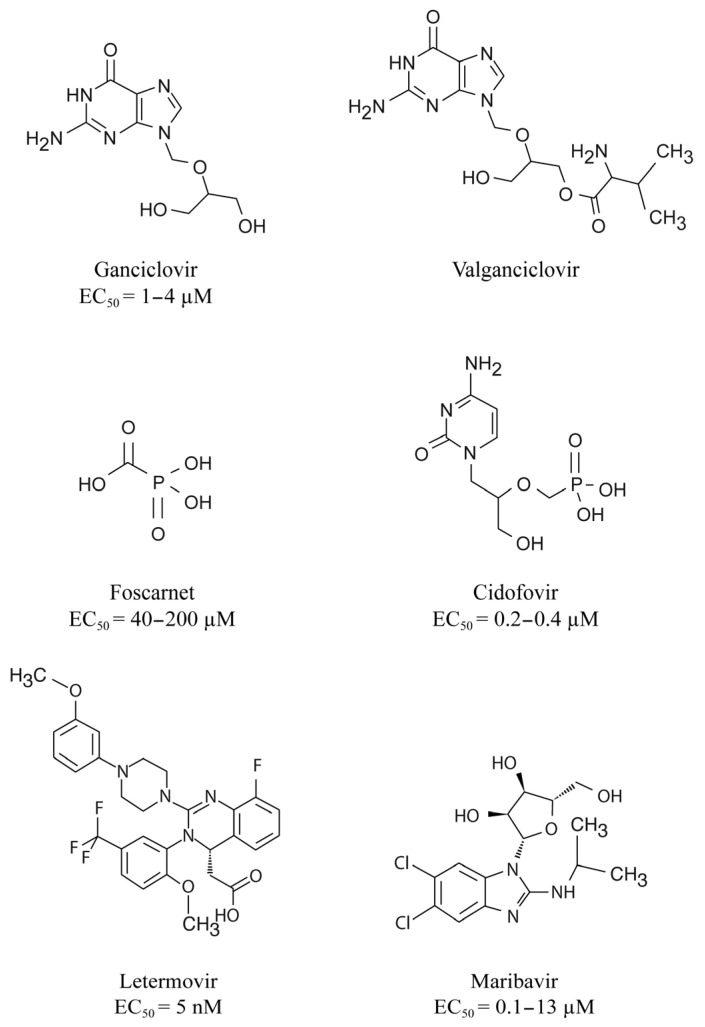
Chemical structures of the different DNA polymerase inhibitors, letermovir and maribavir. Concentrations of antivirals that reduce cytomegalovirus growth by 50% (EC_50_) are also indicated.

Until recently, the prevention and treatment of CMV infection relied on the use of inhibitors of the viral DNA polymerase that is essential for viral replication [6]. The first-line drugs include the nucleoside analog, GCV and its prodrug VGCV whereas the second-line drugs consist in the pyrophosphate analog, FOS and the nucleotide analogue, CDV (Figure 1) [29].

Upon entry into infected cells, GCV is phosphorylated by the viral pUL97 kinase [30] and then converted into its triphosphorylated form by cellular kinases. The active form is a competitive inhibitor of the activity of the viral pUL54 DNA polymerase [31]. GCV-triphosphate also blocks chain elongation following its incorporation into viral DNA [32]. CDV requires only two phosphorylations by cellular kinases to be converted into its active form [33]. CDV-diphosphate is incorporated into viral DNA and prevents chain elongation [34]. FOS does not require any phosphorylation to be active. It directly binds to the pyrophosphate site on the viral DNA polymerase and prevents the incorporation of incoming nucleotides into viral DNA [35].

## 4. Indications for DNA Polymerase Inhibitors

Oral VGCV (900 mg once daily for prophylaxis and twice daily for treatment) and intravenous GCV (5 mg/kg once daily for prophylaxis and twice daily for treatment, dose adjusted for renal function) are indicated in the prevention and in the treatment of active CMV infections. The intravenous formulation of FOS (60 mg/kg every 8 h or 90 mg/kg every 12 h, with a reduction in dose for renal dysfunction) is used for the treatment of CMV retinitis in individuals with acquired immunodeficiency syndrome (AIDS) and infections caused by GCV-resistant CMV in immunocompromised patients. The intravenous formulation of CDV (5 mg/kg once a week for 2 weeks then every 2 weeks) is used for the treatment of CMV retinitis in AIDS patients and is occasionally administered in transplant recipients with drug-resistant CMV infections.

## 5. Prevention and Treatment of CMV Infection 

**Figure 2:**
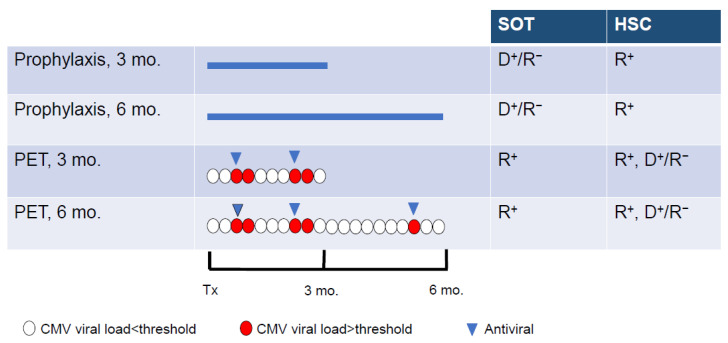
Strategies used for the prevention of CMV infection in solid organ transplant (SOT) and hematopoietic stem cell (HSC) recipients. Universal prophylaxis is based on the administration of antivirals (blue line) to all at-risk patients for 3 or 6 months after transplantation (Tx). During pre-emptive therapy (PET), the antiviral (blue triangle) is administered when the viral load (determined in blood every week for 3 or 6 months) is higher than a defined threshold (red circle) and stopped when the viral is below the threshold (white circle). D^+^/R^−^, donor positive/recipient negative for CMV; R^+^, recipient positive for CMV. Adapted from Limaye et al. [5].

The prevention of active CMV infection is based on two main approaches, universal prophylaxis and pre-emptive therapy (Figure 2) [5]. Universal prophylaxis consists of administering an antiviral agent after the transplantation for a period of 3 or 6 months in the high-risk groups and up to 12 months in lung transplants [19]. The aim of this approach is to maintain viral suppression during the period of the greatest risk for CMV infection or reactivation. Antiviral prophylaxis is effective for the prevention of CMV disease as well as to reduce CMV-associated effects. However, this strategy is associated with a relatively high rate of late-onset CMV diseases following cessation of antiviral administration [4,36] and substantial toxicity. Universal prophylaxis is the main CMV prevention strategy in high-risk SOT recipients. The pre-emptive therapy approach is based on the determination of the viral DNA load every week for 3 or 6 months [5]. The antiviral agent is administered only when the viral DNA load is higher than a defined threshold. Pre-emptive therapy reduces drug exposure and drug-associated toxicity. In the DNA polymerase inhibitors era, pre-emptive therapy was the preferred CMV prevention strategy in HSC recipients to avoid the myelotoxicity of GCV. In order to reduce the risk of delayed-onset CMV diseases after antiviral prophylaxis, a hybrid approach based on the use of prophylaxis during the high-risk periods, i.e., 3 to 6 months after transplantation, followed by a shift to pre-emptive therapy has been also evaluated [37,38]. 

Treatment of initial and recurrent episodes of CMV syndrome and tissue-invasive CMV diseases have been based on the administration of oral VGCV or intravenous GCV [39]. Oral VGCV is preferred for mild to moderate CMV disease and intravenous GCV for life-threatening disease [19]. The viral DNA load should be monitored every week [19]. Antiviral therapy can be stopped at resolution of clinical symptoms and viral clearance in two consecutive samples one week apart. 

## 6. When to Suspect CMV Resistance to Antiviral Drugs?

When the viremia increases or reaches high levels or when clinical symptoms do not resolve despite antiviral therapy, the emergence of drug viral resistance should be suspected [19]. In SOT recipients, exposure to GCV is usually longer than 6 weeks with a median at 5 to 6 months before the emergence of resistance but it can be shorter than 6 weeks in lung transplant recipients.

Prolonged antiviral therapy with inadequate GCV levels is typically associated with the emergence of drug resistance [40]. In SOT recipients, risk factors include the intensity of immunosuppression, a donor positive/recipient negative (D^+^/R^−^) status and lung transplantation [41,42,43]. In HSC recipients, the risk of developing viral drug resistance is increased by a D^−^/R^+^ status, the depletion of T cells, a delayed immune reconstitution and active graft-versus-host disease [44]. The emergence of drug resistance is usually associated with increased morbidity and mortality in transplant recipients.

The incidence of GCV resistance is less than 5–12% in most SOTs but may be as high as 18% in lung transplant recipients [41,45,46] and 31% in intestinal and multi-visceral organ transplants [47,48]. In HSC recipients, the incidence of GCV resistance is usually less than 5% in recipients of an allogeneic graft [49,50] but can be as high as 15% in recipients of a haploidentical graft [51]. 

As FOS and CDV are less frequently used in the clinic, the temporal emergence of CMV strains resistant to these drugs has only been reported in human immunodeficiency virus (HIV)-infected individuals. One small study found an incidence of phenotypic resistance to FOS of 9, 26, 37 and 37% after 3, 6, 9 and 12 months of therapy using an EC_50_ cutoff value of 400 μM (i.e., the concentration of antiviral that reduces CMV growth by 50%) [52]. Another study reported rates of 13, 24 and 37% after 6, 9 and 12 months using an EC_50_ cutoff value of 600 μM [53]. The data on CDV resistance (EC_50_ value ≥ 2–4 μM) seem to indicate a resistance rate similar to those observed with GCV and FOS [52].

## 7. CMV Mutations Conferring Resistance to DNA Polymerase Inhibitors

**Figure 3:**
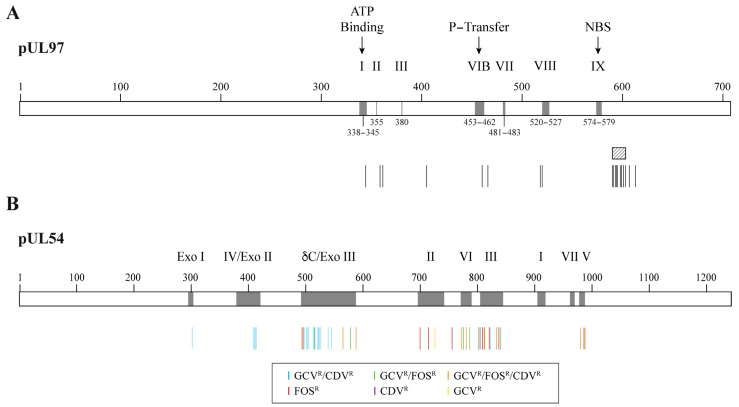
Confirmed cytomegalovirus resistance mutations to DNA polymerase inhibitors. Panel (**A**) shows a representation of the pUL97 kinase with its conserved regions (grey boxes) and the localization of amino acid substitutions conferring resistance to ganciclovir (vertical bars). The ATP-binding site, the phosphate transfer (P-transfer) domain, the nucleoside-binding site (NBS) and some regions conserved among the protein kinase family (i.e., I, II, III, VIB, VII, VIII and IX) are indicated above the boxes. The shaded area corresponds to the codon 590–603 region where different amino acid deletions were identified (i.e., deletions 591–594; 591–607; 595; 595–603; 600 and 601–603). Panel (**B**) shows a representation of pUL54 DNA polymerase with its conserved regions (grey boxes) and the localization of amino acids associated with resistance to ganciclovir (GCV^R^), foscarnet (FOS^R^) and/or cidofovir (CDV^R^) (colored bars). The Roman numbers (I to VII) and δ-region C correspond to conserved regions in the polymerase domain. Exo I, Exo II and Exo III are conserved motifs in the exonuclease domain.

Mutations conferring resistance to GCV initially arise in the pUL97 kinase and impair drug phosphorylation [54]. Mutations conferring resistance to GCV usually emerge at codons 460 and between codons 590 and 607 of the pUL97 kinase (Figure 3A) [55]. Subsequent mutations emerge in the pUL54 DNA polymerase and can confer a high level of resistance and cross-resistance between two or three antiviral drugs [56]. In pUL54 DNA polymerase, drug resistance mutations are widely distributed in the conserved regions of the enzyme (Figure 3B) [55]. GCV and CDV cross-resistant mutations are located in the exonuclease domain and in conserved region V of the polymerase domain. Mutations conferring resistance to FOS or both FOS and GCV are located in conserved regions II, VI and III of the polymerase domain. Mutations in both the pUL97 kinase and pUL54 DNA polymerase result in high levels of resistance to GCV [57,58,59].

## 8. Management of Refractory/Resistant CMV Disease in the DNA Polymerase Inhibitors Era 

Based on the relative increase in their EC_50_ values, UL97 mutations result in insignificant (<2×, low-grade (2–5×) or moderate (5–15×) levels of resistance to GCV (Table 1) [19]. Infection with insignificant or low-grade-resistant UL97 mutants can preferentially be treated with a high dose of intravenous GCV (10 mg/kg twice daily, adjusted for renal function) [19]. Infection with UL97 mutants that are moderately resistant to GCV and UL54 mutants that are susceptible to FOS can be treated with a full dose of FOS (60 mg/kg every 8 h or 90 mg/kg every 12 h, with reduction in dose for renal dysfunction). Infection with UL54 mutants that are resistant to FOS can be treated with CDV (5 mg/kg once a week for 2 weeks and then every 2 weeks) whereas a combination of GCV and FOS at reduced doses [60,61] could be administered in case of resistance to CDV.

## 9. Limitations of the Use of DNA Polymerase Inhibitors

FOS and CDV are only available as intravenous formulations. The administration of FOS or CDV is associated with nephrotoxicity whereas GCV induces myelotoxicity [7,8,9,10]. All these drugs are inhibitors of the viral DNA polymerase with a risk of developing cross-resistance to all agents [6]. Furthermore, several studies have reported a suboptimal outcome in transplant recipients treated with FOS or CDV for refractory/resistant CMV disease [7,8,9,10]. Therefore, there was an urgent need to develop new bioavailable antivirals for the prevention and treatment of CMV infection.

## 10. Novel Antiviral Targets

**Figure 4:**
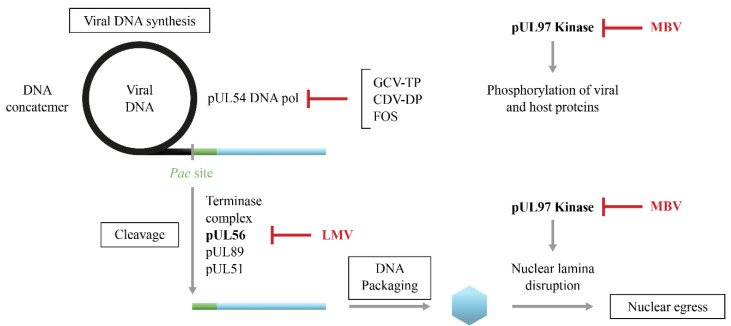
Novel targets for anti-cytomegalovirus inhibitors. During CMV replication, the viral DNA synthesis proceeds by a rolling circle mechanism. This process involves the viral pUL54 DNA polymerase (pol), which is the target of ganciclovir-triphosphate (GCV-TP), cidofovir-diphosphate (CDV-DP) and foscarnet (FOS). The viral terminase complex formed by the pUL56, pUL89 and pUL51 subunits is involved in the cleavage of DNA concatemers at Pac site and their packaging into capsids. Letermovir (LMV) is an inhibitor of the viral terminase complex and more specifically of the pUL56 subunit. On the other hand, pUL97 kinase is involved in the phosphorylation of several viral and host proteins. pUL97 kinase also participates in the disruption of the nuclear lamina and in the nuclear egress of virions. Maribavir (MBV) is a selective inhibitor of the pUL97 kinase activity.

During CMV replication, the viral DNA synthesis proceeds by a rolling circle mechanism (Figure 4) [62]. The viral terminase complex formed by the pUL56, pUL89 and pUL51 subunits [63] is involved in the cleavage of DNA concatemers at the Pac site [64] and their packaging into capsids [65]. LMV is an inhibitor of the viral terminase complex and more specifically of the pUL56 subunit [66]. On the other hand, pUL97 is a serine/threonine protein kinase that phosphorylates itself and several viral and host proteins [15,67]. The pUL97 kinase also participates in the disruption of the nuclear lamina and in the nuclear egress of virions [14]. MBV is a selective inhibitor of the pUL97 kinase activity [68,69].

## 11. Letermovir, a Viral Terminase Inhibitor

LMV is a dihydroxyquinazoline derivative (Figure 1) that demonstrates in vitro activity against CMV with an EC_50_ value in the nanomolar range but it is not active against other herpesviruses [70,71]. LMV is a specific inhibitor of the CMV terminase complex and shows activity against isolates resistant to DNA polymerase inhibitors [66,71,72]. LMV interferes with the cleavage of the viral DNA and its packaging into capsids (Figure 4) [66].

LMV can be administered orally or intravenously (480 mg once daily for up to 12 weeks or 240 mg if given with cyclosporin). The intravenous formulation is intended to be used immediately after transplantation as well as in patients with gastrointestinal complications that make the ingestion and absorption of oral drugs difficult [73]. LMV is safe and well tolerated [74]. In contrast to GCV, the administration of LMV is not associated with myelotoxicity, which allows its use in prophylaxis strategy for the prevention of CMV infection in HSC recipients. As LMV targets the viral terminase complex, there is no risk of cross-resistance with other antiviral drugs [74]. LMV affects cytochrome P isoenzymes and transporters suggesting that drug interactions should be anticipated with immunosuppressors (e.g., tacrolimus, cyclosporine A) and fungicides (e.g., posaconazole, voriconasole) [75,76].

LMV was approved under the trade name, Prevymis^®^ (Merck Sharp & Dohme LLC, Rahway, NJ, USA), for the prophylaxis of CMV infection in adult R^+^ allogeneic HSC recipients [77]. This approval was based on a pivotal phase 3 study that showed that clinically significant CMV infections occur in 19.1% of patients in the LMV group compared to 50% in the placebo group (*p* < 0.001) at week 14 after treatment [78]. Clinically significant CMV infection was reduced to 37.5% of patients in the LMV group compared to 60.5% in the placebo group (*p* < 0.001) at week 24 after treatment Furthermore, all-cause mortality was reduced from 25.5% in the placebo group compared to 20.9% in the LMV group (*p* = 0.12) at week 48 after treatment [78]. The extension of the pivotal phase 3 trial (NCT03930615) from day 100 to day 200 after LMV prophylaxis in R^+^ of an allogeneic HSC is now terminated. A systematic review and meta-analysis of observational studies reported that LMV was effective in reducing the risk of CMV-related complications [i.e., CMV reactivation (*p* < 0.05), clinically significant infection (*p* < 0.05) and CMV disease (*p* < 0.05) by day 100 and day 200 after allogeneic HSC] and mortality [i.e., all-cause (*p* < 0.01) and non-relapse (*p* < 0.01) mortality] after day 200 post-transplantation [79].

The approval of LMV is changing the prevention of CMV infection in HSC recipients, which was mainly based on pre-emptive therapy due to the myelotoxicity associated with VGCV/GCV. Further investigations are still needed to evaluate the efficacy of LMV prophylaxis in R^+^ pediatric patients who received an allogeneic HSC (NCT03940586) as well as in SOT recipients. A phase 3 trial (NCT0344869) evaluated the efficacy and safety of LMV compared to VGCV for the prevention of CMV disease in D^+^/R^−^ kidney transplant recipients through week 52. LMV was non-inferior to VGCV for the prevention of CMV disease (10.4% vs. 11.8%) and was associated with a lower rate of leukopenia or neutropenia through week 28 [80]. Further studies are planned to evaluate the efficacy and safety of LMV for CMV prevention in lung transplant (NCT05041426), heart transplant (NCT04904614) and thoracic transplant (NCT06066957) recipients.

## 12. CMV Resistance to Letermovir

**Figure 5:**
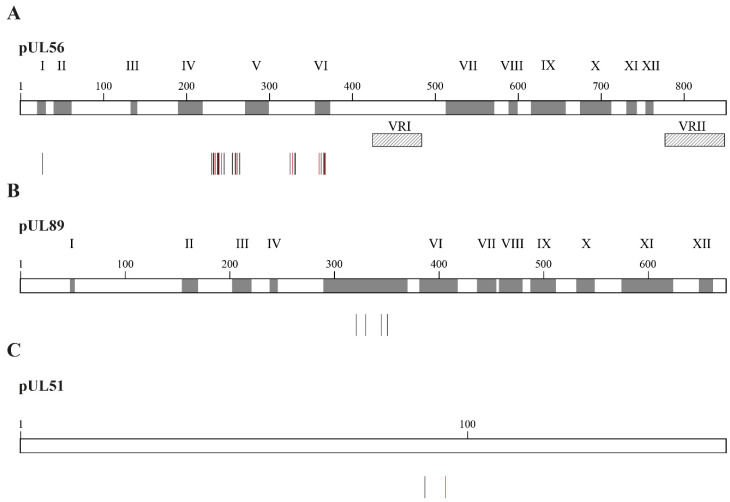
Confirmed amino acid changes associated with resistance to letermovir detected in CMV laboratory strains and clinical specimens. Panel (**A**) shows amino acid substitutions in the pUL56 subunit associated with letermovir resistance (vertical bars). Grey boxes represent the conserved regions of pUL56, which are numbered I to XII. Hatched boxes represent the two variable regions, which are labeled as VRI and VRII. Panel (**B**) shows amino acid substitutions in the pUL89 subunit conferring resistance to letermovir (vertical bars). Grey boxes represent the conserved regions in pUL89, which are numbered I to XII. Panel (**C**) shows amino acid substitutions in pUL51 subunit conferring letermovir resistance (vertical bars). In all panels, vertical bars show amino acid substitutions associated with letermovir resistance identified in laboratory strains (black) and clinical specimens (red).

Mutations selected under LMV were detected in the three subunits of the viral terminase complex, pUL56, pUL89 and pUL51 [74] (Figure 5). Mutations conferring resistance to LMV in the pUL56 subunit are located at codon 25 and between codons 229 and 369 (Figure 5A) [74]. The levels of resistance to LMV may vary from two to absolute resistance for mutations located at codon 325 of the pUL56 subunit (Table 2). Combinations of two or three mutations in the *UL56* gene result in markedly increased levels of LMV resistance [81,82,83]. All mutations identified in the pUL89 subunit are located in conserved region V of the protein (Figure 5B) and were associated with low-grade resistance to LMV (Table 2). To date, only two mutations were described in the pUL51 subunit (Figure 5C); one detected in a clinical specimen conferred a 13.8-fold increase in EC_50_ value (Table 2) [84]. Mutations in both the pUL51 and pUL56 subunits were synergistic, whereas mutations in both the pUL89 and pUL56 subunits were additive [85]. In vitro studies showed that LMV resistance mutations emerged at a lower selection passage compared to FOS and GCV, suggesting that LMV may have a lower genetic barrier to resistance [81].

## 13. Disadvantages of Letermovir

LMV is highly specific to CMV and does not possess activity against other herpesviruses such as herpes simplex virus and varicella zoster virus, which may require the use of additional antiviral drugs for the prevention or treatment of these infections. LMV has a potentially low genetic barrier to the emergence of resistance, with single mutations that can be associated with very high levels of resistance [86]. The emergence of resistance to LMV should be thus monitored early in patients with a virologic failure. The rate of LMV resistance after prophylaxis is low and comparable to those of DNA polymerase inhibitors. However, it is anticipated that the rate of LMV resistance could be higher when used in treatment. Therefore, LMV is not currently investigated as a treatment option, except in a small phase 2 study (NCT03728426). LMV was used off-label as salvage therapy for refractory/resistant CMV diseases [87], but MBV is now a better option in that situation. 

## 14. Maribavir, a pUL97 Kinase Inhibitor

Maribavir is a benzimidazole-L-riboside derivative (Figure 1) that demonstrated in vitro activity against CMV including strains resistant to GCV [88], Epstein–Barr virus [89] and human herpesvirus 6 but not against herpes simplex virus and varicella zoster virus. Maribavir is a selective inhibitor of the pUL97 kinase [68,69]. It prevents the phosphorylation of viral and host proteins and the nuclear egress of virions (Figure 4) [90].

In contrast to FOS and CDV, MBV is available as an oral formulation, which may thus facilitate the treatment of patients with refractory/resistant CMV diseases. The dosage of oral MBV is 400 mg twice daily. MBV is safe and well tolerated. It could be administered to patients with an underlying kidney dysfunction and/or myelosuppression. Due to its lack of myelotoxicity, MBV may have some advantages over VGCV for use as CMV prophylaxis. However, it is not recommended to use MBV for the treatment of retinitis and encephalitis due to its low penetration in the eyes and brain [91]. The use of MBV as initial treatment for CMV infection requires further investigations to evaluate the risk of cross-resistance with GCV as well as the rate of emergence of MBV resistance. 

MBV was approved under the trade name Livtencity^®^(Takeda^®^ Pharmaceutical Company Ltd., Osaka, Japan) for the treatment of adult and pediatric patients with post-transplant CMV infection/disease refractory/resistant to treatment with DNA polymerase inhibitors [92]. This approval was based on the results of the pivotal SOLTICE trial (NCT02931539) that shows CMV clearance in 55.7% of patients in the MBV group compared to 23.9% in the investigator-assigned therapy group (*p* < 0.001) after week 8. CMV clearance and symptom control were observed in 18.7% of patients in the MBV group compared to 10.3% of patients in the investigator-assigned therapy group (*p* < 0.01) from week 9 to week 16 [93]. Clinically relevant recurrences were also lower in the MBV group. All-cause mortality was similar between groups.

A phase 3 study (NCT02927067) evaluating the efficacy of MBV compared to VGCV for the treatment of CMV infection in HSC recipients is completed but the results are not yet published. Furthermore, a phase 3 study (NCT05319353) evaluating the safety and tolerability, pharmacokinetics and antiviral activity of MBV for the treatment of CMV infection in children and adolescents who received a SOT or a HSCT is planned.

## 15. Resistance of CMV to Maribavir

**Figure 6:**
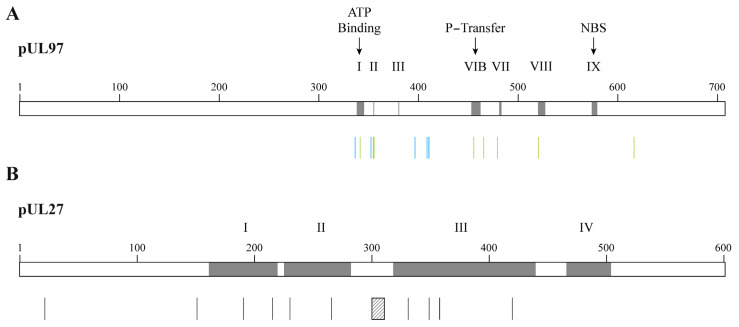
Confirmed amino acid changes associated with resistance to maribavir detected in CMV laboratory strains and clinical specimens. Panel (**A**) shows amino acid substitutions in the pUL97 kinase conferring CMV resistance to maribavir (vertical bars in blue) or to maribavir/ganciclovir (vertical bars in green). Grey boxes represent the ATP-binding site, the phosphate transfer (P-transfer) domain, the nucleoside-binding site (NBS) and some regions conserved among the protein kinase family (i.e., I, II, III, VIB, VII, VIII and IX). Panel (**B**) shows amino acid substitutions in the pUL27 associated with resistance to maribavir (vertical bars in black). The hatched box represents codons 301–311 deletion that confers maribavir resistance. Grey boxes represent the conserved regions in pUL27, which are numbered I to IV.

Mutations conferring resistance to MBV only are located in the vicinity of the ATP-binding site of pUL97 kinase [74] (Figure 6A). Mutations cross-resistant to MBV and GCV were detected in the P-loop and at distant sites from it. UL97 mutants can demonstrate very high levels of resistance to MBV, such as mutations located at codons 409 and 411 and mutation C480F, which confers cross-resistance to GCV and MBV (Table 3). Dual mutations in the pUL97 kinase result in a synergistic increase in MBV EC_50_ values [94]. Compensatory mutations also emerged in the *UL27* gene under MBV and confer low levels of resistance to the drug [74]. Mutations conferring resistance to MBV were widely distributed in UL27 protein and were found in conserved regions I, II and III (Figure 6B). Combined mutations in *UL97* and *UL27* genes result in a two-fold increase in MBV EC_50_ values [95].

## 16. Disadvantages of Maribavir

The use of MBV is limited by the possible cross-resistance phenotype with GCV [96]. MBV seems to possess an intermediate genetic barrier to resistance compared to LMV (lower) and DNA polymerase inhibitors (higher), but further investigations are still needed. MBV lacks activity against herpes simplex and varicella zoster viruses and the use of additional antiviral agents may thus be required to treat these infections. The levels of immunosuppressors such as tacrolimus and sirolimus need to be monitored due to possible drug interactions with MBV [97,98].

## 17. Drug Combinations

Several antiviral drugs with different mechanisms of action are now available, which opens the door to drug combinations. Combination therapy may increase the therapeutic effect, reduce toxicity and prevent the selection of drug-resistant isolates [99]. In vitro studies have shown that combinations of LMV with GCV and CDV have additive effects, whereas additive and minor synergistic effects are seen with FOS [100]. The combination of LMV and artesunate results in moderate synergistic effects [101] and MBV combined with artesunate has synergistic effects in vitro [102]. As MBV inhibits the phosphorylation of GCV by pUL97 kinase, this drug combination leads to antagonistic effects [103]. Combinations of MBV with FOS, CDV and LMV result in synergistic effects, whereas strongly synergistic effects are seen when combining MBV with rapamycin, a mammalian target of rapamycin (mTOR) inhibitor [104]. Furthermore, combinations of LMV and MBV were shown to be synergistic [105].

## 18. New Perspectives for the Prevention and Treatment of CMV Infection/Disease

The characteristics of anti-CMV drugs approved for the prevention and treatment of CMV infection and disease are summarized in Table 4. In contrast to GCV, LMV is not associated with myelotoxicity. LMV is thus replacing GCV for CMV prophylaxis in HSC recipients. LMV could also be an option for CMV prophylaxis in SOT. The efficacy and safety of LMV for CMV prophylaxis in SOT recipients is thus further evaluated in clinical trials.

In contrast to FOS and CDV, MBV can be administered orally and is associated with fewer side effects. MBV is thus replacing FOS and CDV for the treatment of refractory/resistant CMV diseases. The efficacy and safety of MBV as a primary treatment option for CMV diseases in SOT and HSC recipients need to be further evaluated in clinical trials, especially the risk for cross-resistance with GCV and the genetic barrier to resistance. 

## 19. Conclusions

There have been important advances in the prevention and treatment of CMV infections over the last 5 years with the approval of LMV and MBV. The administration of both drugs is not associated with myelotoxicity or other serious side effects as seen with DNA polymerase inhibitors. LMV and MBV target other viral proteins than the pUL54 DNA polymerase with a low risk for cross-resistance between antiviral agents, especially with LMV. The use of antiviral combinations could be also envisaged as a strategy to reduce toxicity and the emergence of drug resistance in high-risk patients. However, the genetic barrier to resistance of these two drugs is not well characterized, which requires further investigations. For now, the two novel antiviral agents occupy specific niches in the armamentarium of drugs available for the prevention and treatment of CMV infections. Further studies evaluating the benefits of these drugs for other patient populations or other prevention or treatment approaches should be available soon. The search for novel compounds also remains a high priority to broaden the range of antiviral agents available for the prevention and treatment of CMV infections.

## Figures and Tables

**Table 1:** Relative levels of ganciclovir resistance of CMV UL97 mutants.

Genotype Frequency	Relative Increase in EC_50_ Value Compared to Wild Type		
	<2× (Insignificant)	2–5× (Low-Grade)	5–15× (Moderate)
Most common		C592G	M460I/V, H520Q, A594V, L595S, C603W
Less common at codons 460, 590–607	E596D, N597D, K599E/R, L600I, T601M, C603S, D605E, C607F	A591V, A594E/T/S, E596G/Q, C603S, E596G, 600del2, C607F	M460T, A594G/P, 595del, L595F/W/del, E596Y, 597del2, 599del, K599T, 600del, 601del, 601del2, C603R, C607Y, del(≥3)
Atypical loci	M615V, Y617H, A619V, L634Q, E655K, A674T	K359E/N/Q, E362D, L405P, I610T, A613V	F342S/Y, K355M, V356G, V466G, C480R, C518Y, P521L

All amino acid substitutions or deletions (del) were detected in clinical specimens and were confirmed by recombinant phenotyping.

**Table 2:** Relative levels of letermovir resistance in CMV mutants.

Genes	Relative Increase in EC_50_ Value Compared to Wild Type			
	1.8–4.9×	5–19×	20–99×	>100×
*UL56*	S229F, V231A, **Q234R**, V236A, T244K/R, L254F, L257I, F261C/L, Y321C, L321C, L328V, M329T, **V363I**, A365S, N368D, **R369K**	C25F, **V231L**, **N232Y**, V236L, **E237D/G**, **L257F**, K258E, R369M	**V236M**, **R369G/S/T**	L241P, **C325F/R/W/Y**
*UL89*	N320H, N329S, D344E, T350M			
*UL51*	P91S	**A95V**		

Amino acid substitutions indicated in bold were detected in clinical specimens and were confirmed by recombinant phenotyping.

**Table 3:** Relative levels of maribavir and/or ganciclovir resistance of CMV UL97 mutants.

Drugs	Relative Increase in EC_50_ Value Compared to Wild Type			
	1.8–4.9×	5–19×	20–99×	>100×
Maribavir	F342Y, L337M	F342S, V353A, H411N/Y	T409M, H411L	K355del, V356G, L397R, D456N, V466G, C480F, C480R, P521L, Y617del
Ganciclovir	C480F	F342S, F342Y, K355del, V356G, D456N, V466G, C480R, P521L, Y617del		

Amino acid substitutions that are underlined were detected in clinical specimens and were confirmed by recombinant phenotyping. Amino acid substitutions or deletions (del) conferring resistance to maribavir only and to maribavir/ganciclovir are shown in blue and green, respectively.

**Table 4:** Characteristics of approved anti-CMV drugs.

Parameters	GCV/VGCV	FOS	CDV	LMV	MBV
Class of antiviral	Nucleoside analogue	Pyrophosphate analogue	Nucleotide analogue	Dihydroxyquinazoline	Benzimidazole L-riboside
Viral target	DNA pol (pUL54)	DNA pol (pUL54)	DNA pol (pUL54)	Terminase complex (pUL56)	Viral kinase (pUL97)
Activation	pUL97 and cellular kinases	Not required	Cellular kinases	Not required	Not required
In vitro antiviral activity	Herpesviruses and hepatitis B virus	All herpesviruses	Most DNA viruses	CMV only	CMV, EBV, HHV-6 but not HSV and VZV
EC_50_ values against CMV	1–4 μM	40–200 μM	0.2–0.4 μM	5 nM	0.1–13 μM
Resistance gene(s)	*UL97* *UL54*	*UL54*	*UL54*	*UL56* *UL89* * UL51	*UL97* *UL27* ^#^
Activity against drug-resistant viruses	Risk of cross-resistance with other DNA pol inhibitors and MBV	Risk of cross-resistance between DNA pol inhibitors	Risk of cross-resistance between DNA pol inhibitors	No risk of cross-resistance	Possible cross-resistance with GCV
Genetic barrier to resistance	High	High	High	May be low	May be intermediate
Level of drug resistance	Low to intermediate	Low to intermediate	Low to intermediate	Low to high	Low to high
Oral bioavailability	6% (GCV) 60% (VGCV)	12% to 22%	2% to 26%	35%	>90%
Half-life (t_1/2_)	2.5 h to 3.6 h	3.4 h to 5 h	24 h and 65 h for first and second elimination half-lives, respectively	10 h	3 h to 5 h
Protein binding	1% to 2%	14% to 17%	6%	99%	98%
Excretion	Renal	Renal	Renal	Biliary	Renal (61%; mainly metabolized drug) and biliary (14%; unchanged and metabolized drug)
Route of administration	iv, ocular implant (GCV) po (VGCV)	iv	iv	po, iv	po
Dosing	5 mg/kg (GCV) 900 mg (VGCV) qd (prophylaxis) bid (treatment)	60 mg/kg tid 90 mg/kg bid	5 mg/kg once a wk for 2 wks, then every 2 wks	480 mg qd	400 mg bid
Toxicity	Myelosuppression	Renal dysfunction, electrolyte disturbances, hematologic toxicities	Nephrotoxicity	Safe and well tolerated	Safe and well tolerated
Drug interactions	Some anti-HIV drugs	Nephrotoxic drugs	Nephrotoxic drugs	Immunosuppressive drugs and fungicides	Immunosuppressive drugs

Key: *, To date, mutations conferring resistance to letermovir in *UL89* gene were found in laboratory strains only. ^#^, To date, mutations conferring resistance to maribavir in *UL27* gene were found in laboratory strains only. bid, twice a day; CDV, cidofovir; CMV, cytomegalovirus; EBV, Epstein–Barr virus; EC_50_, concentration of antivirals that decrease viral growth by 50% in vitro; FOS, foscarnet; GCV, ganciclovir; HIV, human immunodeficiency virus; HSV, herpes simplex virus; HHV-6, human herpes virus 6; iv, intravenous; LMV, letermovir; MBV, maribavir; po, per os; qd, once daily; tid, three times a day; VGCV, valganciclovir; VZV, varicella zoster virus; wk, week. Data are from references [106,107,108,109,110,111].

## Data Availability

Data is contained within the article or supplementary material.
